# Supplementary material for: Human genome-wide RNAi screen reveals host factors required for enterovirus 71 replication
Source: Nat Commun. 2016 Oct 17;7:13150. doi: 10.1038/ncomms13150 (PMC5071646; doi:10.1038/ncomms13150)
Supplement: Supplementary Information — Supplementary Figures 1 - 7 [file ncomms13150-s1.pdf]

Supplemental Figures

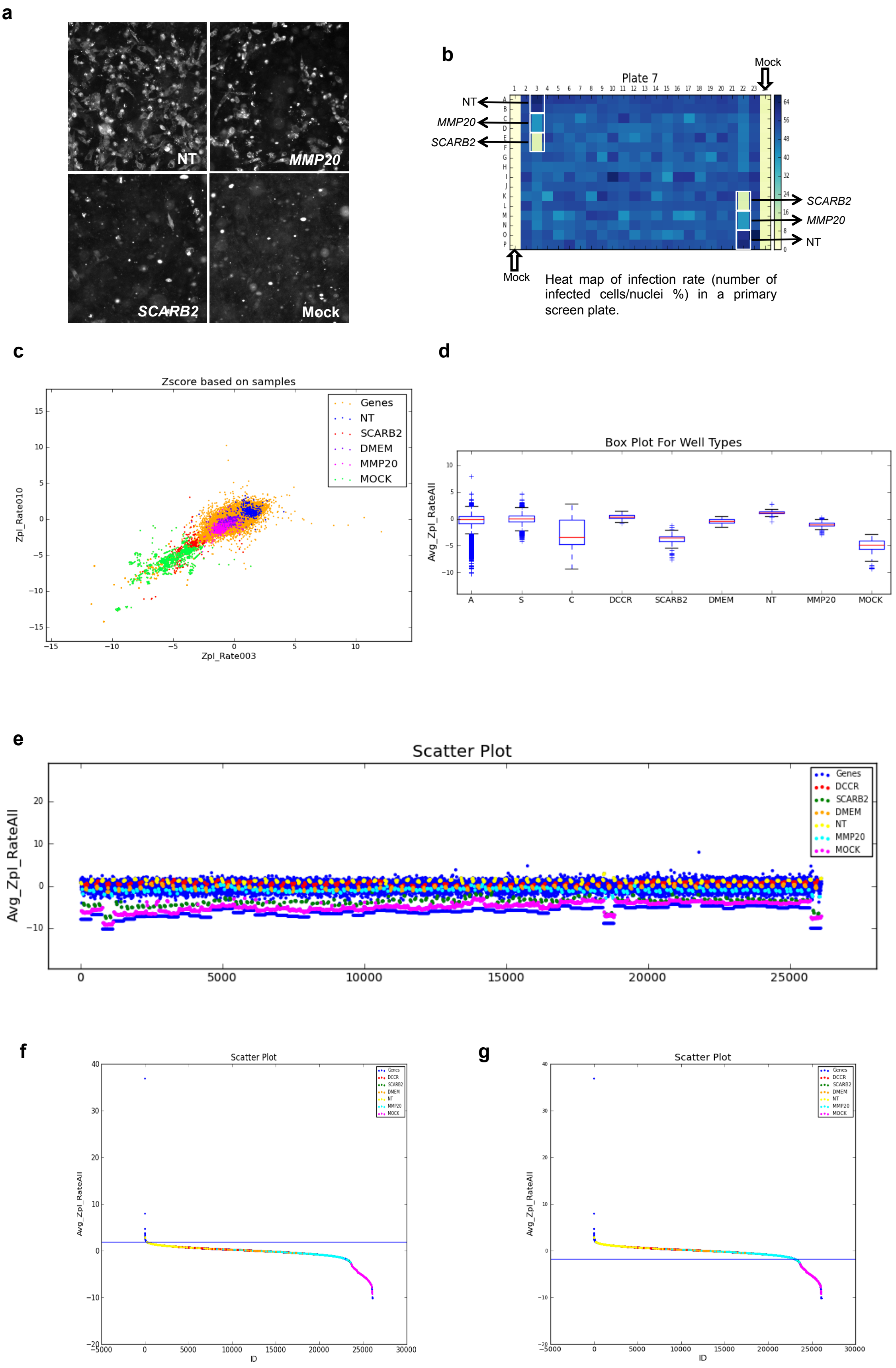

Supplementary Figure 1. Quality checks and controls for genome-wide screen. (a) Representative gray scale images of controls acquired during primary screening. The images were taken in the FITC channel and signal indicates expression of viral protein VP0/VP2. (**b**) Sample plate layout showing a heat map based on infection rate of each well with indicated well positions for controls. **(c)** Correlation of all samples and controls from two independent screens. **(d)** Boxplot showing median, quartiles and outliers for data from three independent screens, A=all, S=sample/gene, C=all controls and the individual controls are shown. **(e)** Scatter plot showing distribution of mean Z values from three independent screens with controls highlighted. The same data is shown in a ranked score distribution to show hit cutoff for HRFs-above blue line (**f**) and HSFs-below blue line **(g).**

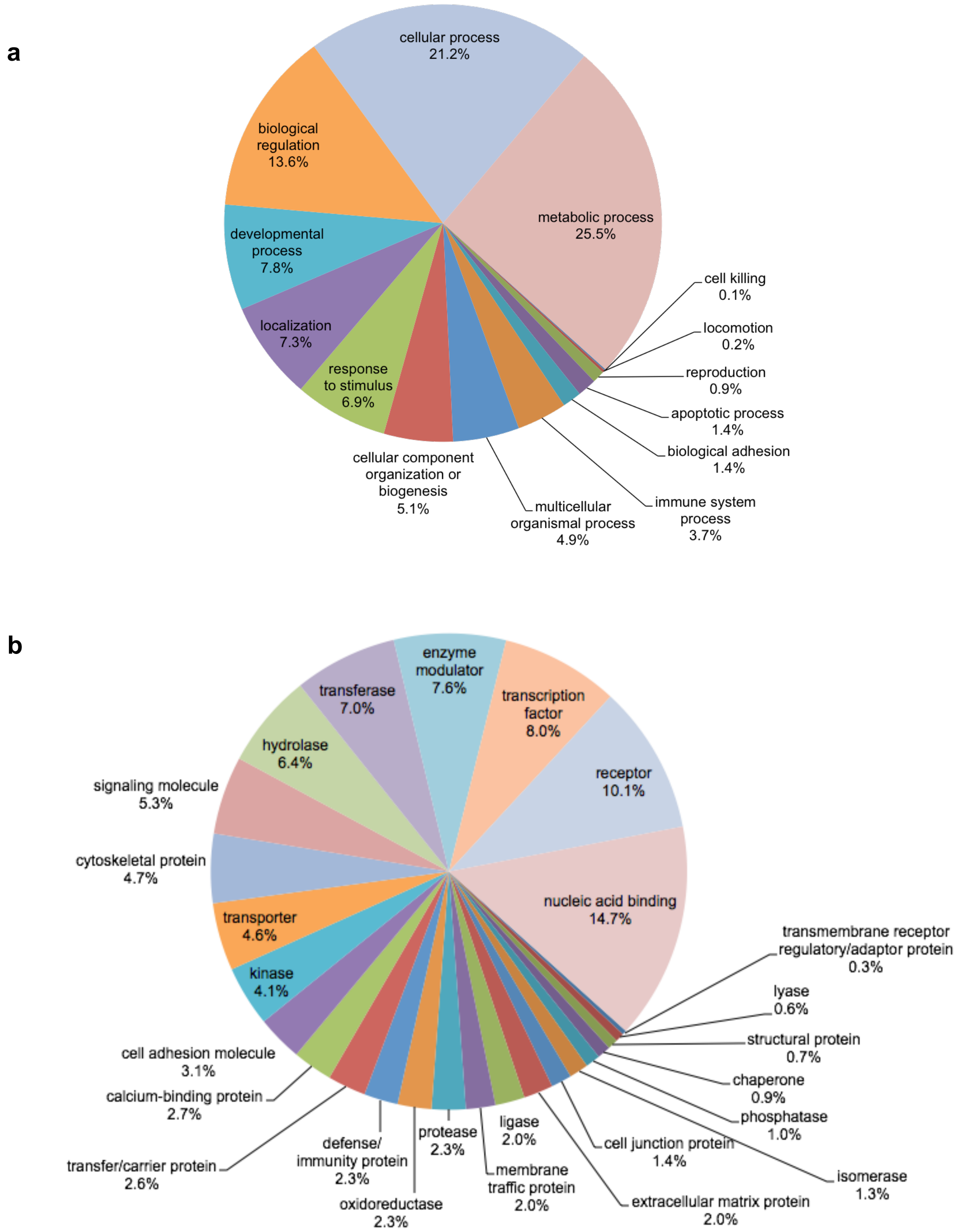

Supplementary Figure 2. Functional classifications of screening hits. Pie charts representing the functional distribution of 635 classifiable hits based on Gene Ontology (GO) database. Hits were classified based on (**a**) bioprocess and (**b**) protein class.

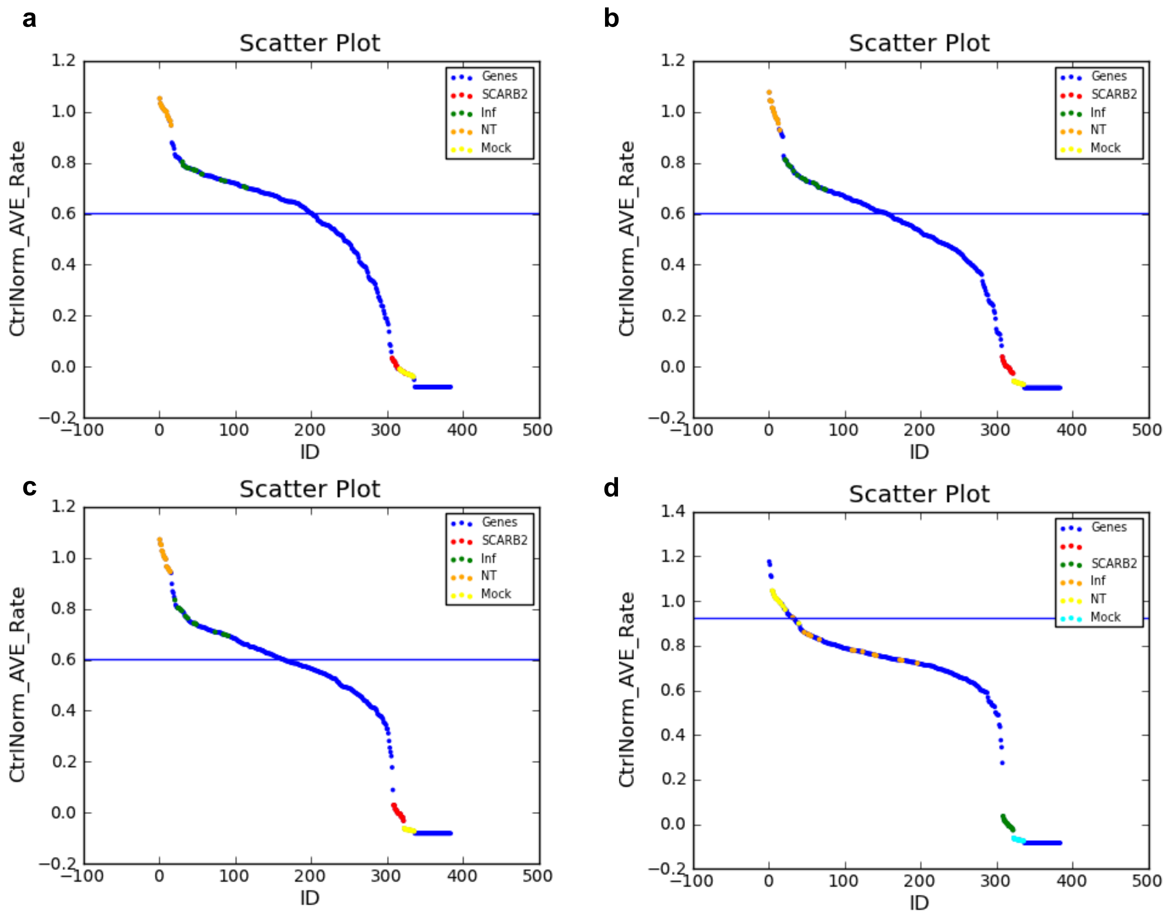

Supplementary Figure 3. Hit confirmation from secondary validation screen. The 517 genes selected for ON-TARGETplus validation were arrayed into 4 plates with all the HRFs in plate 4 **(d).** Scatter plots of the normalized rates are shown for plate 1-4 in (**a-d**) respectively. Blue horizontal lines indicate cutoffs for hit confirmation with $Ctrl_{NORM}<0.60$ for HSFs (**a-c**) and $Ctrl_{NORM}>0.92$ for HRFs (**d**) based on observations of data distribution.

a

Hit	Ave #1	Ave #2	Ave #3	Ave #4
RASSF4	94.20	108.32	101.76	137.28
AURKB	111.23	121.44	120.60	116.07
CDK6	114.57	133.75	128.12	118.64
TGFB1I1	100.40	87.20	124.78	118.08

HSFs	Ave #1	Ave #2	Ave #3	Ave #4
SMARCD2	79.37	103.15	73.24	94.50
PMVK	94.35	69.54	97.50	101.44
DTX1	90.12	74.20	85.87	97.90
SNUPN	98.85	95.60	95.95	95.11
MYH4	92.82	102.23	17.61	96.61
RDX	94.79	69.29	94.53	45.82
PAK1	91.42	100.18	99.94	93.67
LMO1	88.85	90.59	90.28	94.08
CNOT3	91.58	69.24	81.76	79.73
CIZ1	51.51	39.79	100.87	96.76
GRIN2A	111.47	72.06	103.49	83.02
GOT1L1	82.28	94.34	99.05	98.68
CD1A	50.78	94.18	84.62	104.86
IFNGR2	55.27	89.59	86.32	100.45
CCR4	90.91	98.89	96.75	59.72
OR5K3	127.76	91.99	89.67	101.65
PCDHAC1	90.15	91.31	80.40	94.90
NGLY1	30.01	101.03	106.59	103.14
KLK13	86.85	107.14	68.62	94.26

140.00
130.00
120.00
110.00
100.00
90.00
80.00
70.00
60.00
50.00
40.00
30.00
20.00
10.00
0.00

c

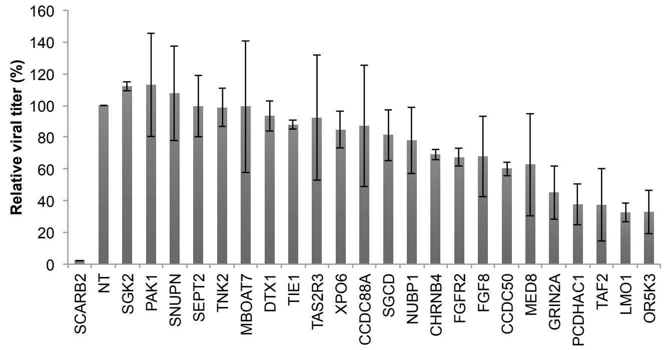

Gene	*p* value
SCARB2	1.94E-03
NT	
LPHN2	5.93E-02
HSD17B12	7.27E-02
NRIP2	1.85E-01
SMARCD2	2.74E-02
CCR4	8.74E-03
CIZ1	3.26E-02
RNMT	7.88E-02
IL7	7.15E-03
TBP	1.84E-02
CNOT3	2.31E-02
KLK13	4.01E-02
CLRN1	1.19E-01
IFNGR2	6.84E-03
PMVK	3.97E-03
GOT1L1	4.36E-03
RDX	2.95E-02
NGLY1	4.78E-02
CD1A	1.87E-02
MYH4	2.21E-03

b

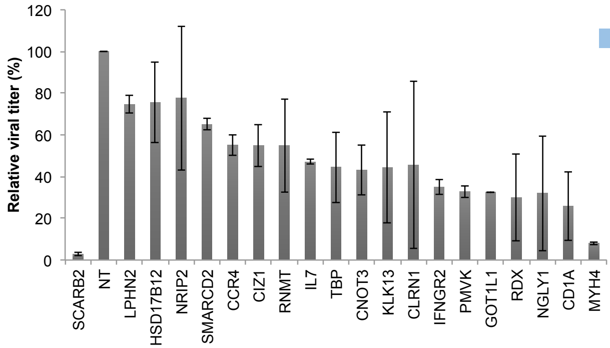

Gene	*p* value
SCARB2	7.14E-07
NT	
SGK2	1.50E-02
PAK1	3.15E-01
SNUPN	3.75E-01
SEPT2	4.88E-01
TNK2	4.42E-01
MBOAT7	4.92E-01
DTX1	2.11E-01
TIE1	1.50E-02
TAS2R3	4.02E-01
XPO6	1.00E-01
CCDC88A	3.41E-01
SGCD	1.23E-01
NUBP1	1.38E-01
CHRNB4	2.88E-03
FGFR2	7.44E-03
FGF8	1.07E-01
CCDC50	3.06E-03
MED8	1.23E-01
GRIN2A	2.19E-02
PCDHAC1	1.03E-02
TAF2	2.97E-02
LMO1	1.84E-03
OR5K3	1.02E-02

d

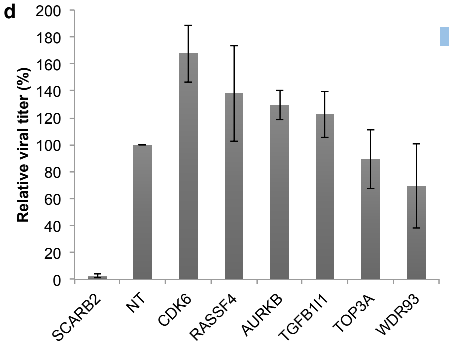

Gene	*p* value
SCARB2	4.47E-05
NT	
CDK6	2.28E-02
RASSF4	1.34E-01
AURKB	3.04E-02
TGFB1I1	1.01E-01
TOP3A	2.74E-01
WDR93	1.49E-01

Supplementary Figure 4. Deconvolution of validated hits and plaque reduction assays. **(a)** Select genes from the validated hit lists were deconvoluted to select for single siRNA sequence for subsequent use in downstream experiments. Heatmap shows the relative infection rate for each sequence and results are from two-independent experiments. **(b)** and **(c)** show NT-normalized viral titers from EV71-infected cells (MOI 1, 12hpi) after the knockdown of select HSF genes while **(d)** show that for HRFs. Results are from two independent experiments and error bars represent standard deviations. p-values shown are for one tailed, Student's T-Test performed for each sample against NT controls.

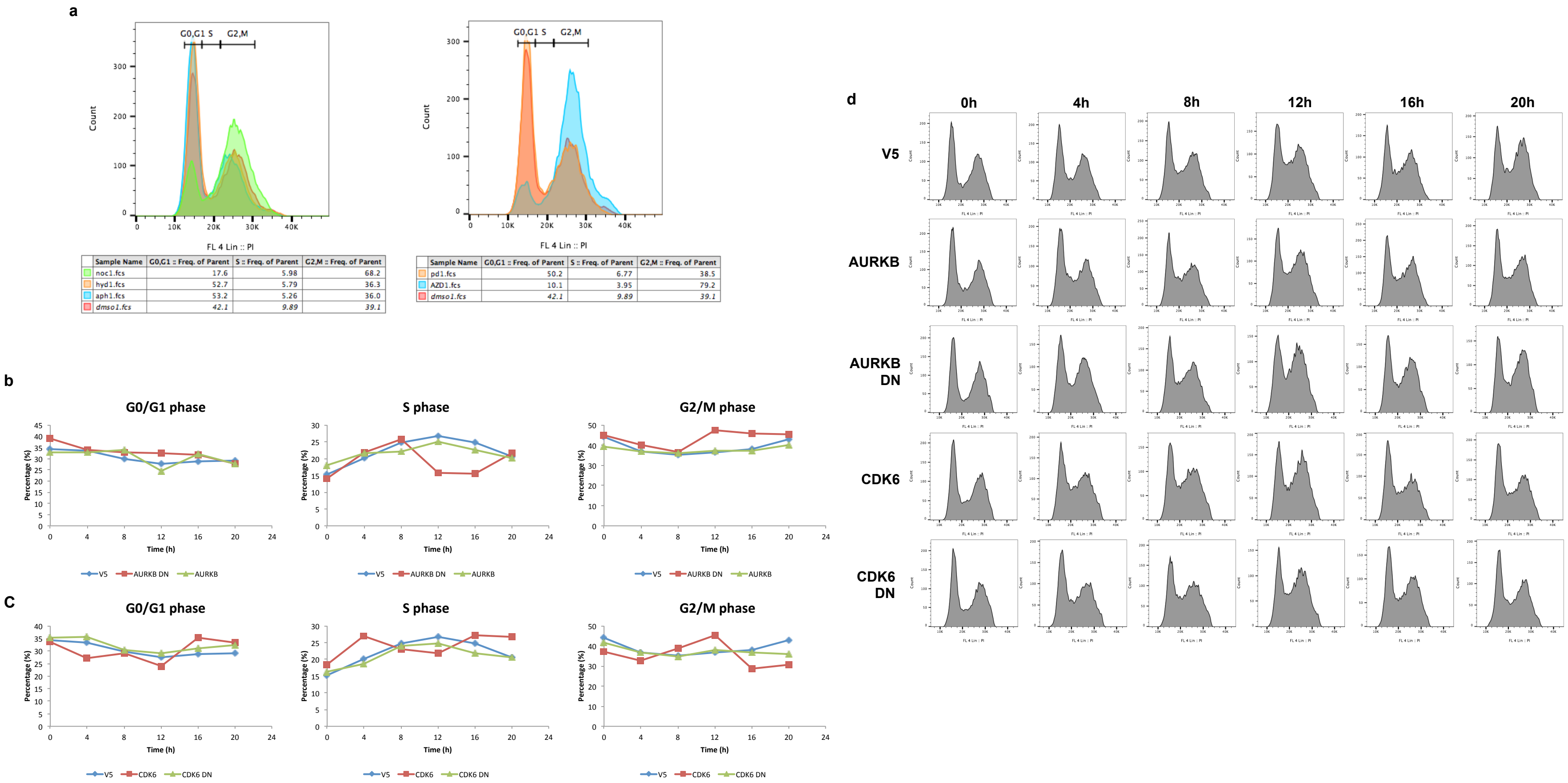

	Sample Name	G0,G1 :: Freq. of Parent	S :: Freq. of Parent	G2,M :: Freq. of Parent
	noc1.fcs	17.6	5.98	68.2
	hyd1.fcs	52.7	5.79	36.3
	aph1.fcs	53.2	5.26	36.0
	dmso1.fcs	*42.1*	*9.89*	*39.1*

	Sample Name	G0,G1 :: Freq. of Parent	S :: Freq. of Parent	G2,M :: Freq. of Parent
	pd1.fcs	50.2	6.77	38.5
	AZD1.fcs	10.1	3.95	79.2
	dmso1.fcs	*42.1*	*9.89*	*39.1*

Supplementary Figure 5. Cell cycle perturbations in compound-treated and overexpression cell lines. (**a**) RD cells were treated with nocodazole (5μg/ml), aphidicolin (10μg/ml), hydroxyurea (5mM), AZD1152-HQPA (100nM), PD0332991 (10μM) or 0.1% DMSO in the same manner as described for Fig. 3g before fixation and staining with propidium iodide (PI) for flow cytometry analysis. Histograms of gated, PI-stained cells are shown for each treatment with 0.1% DMSO serving as the reference control. The percentages of cells in G0/G1, S or G2/M phase for each sample are also tabulated below the overlaid histograms. AURKB, AURKB DN, CDK6, CDK6 DN or V5 (empty vector) overexpressing stable cell lines were serum-starved for 16h before replacement with DMEM supplemented with 10% FCS. Samples were then harvested, fixed and stained with PI at the indicated timepoints before analysis via flow cytometry. The percentages of cells in G0/G1, S or G2/M phase are shown for AURKB and AURKB DN in (**b**) and CDK6 and CDK6 DN in (**c**) while (**d**) shows the complete histograms of gated PI populations at each timepoint for all the cell lines evaluated. Results shown here are representative of three independent experiments.

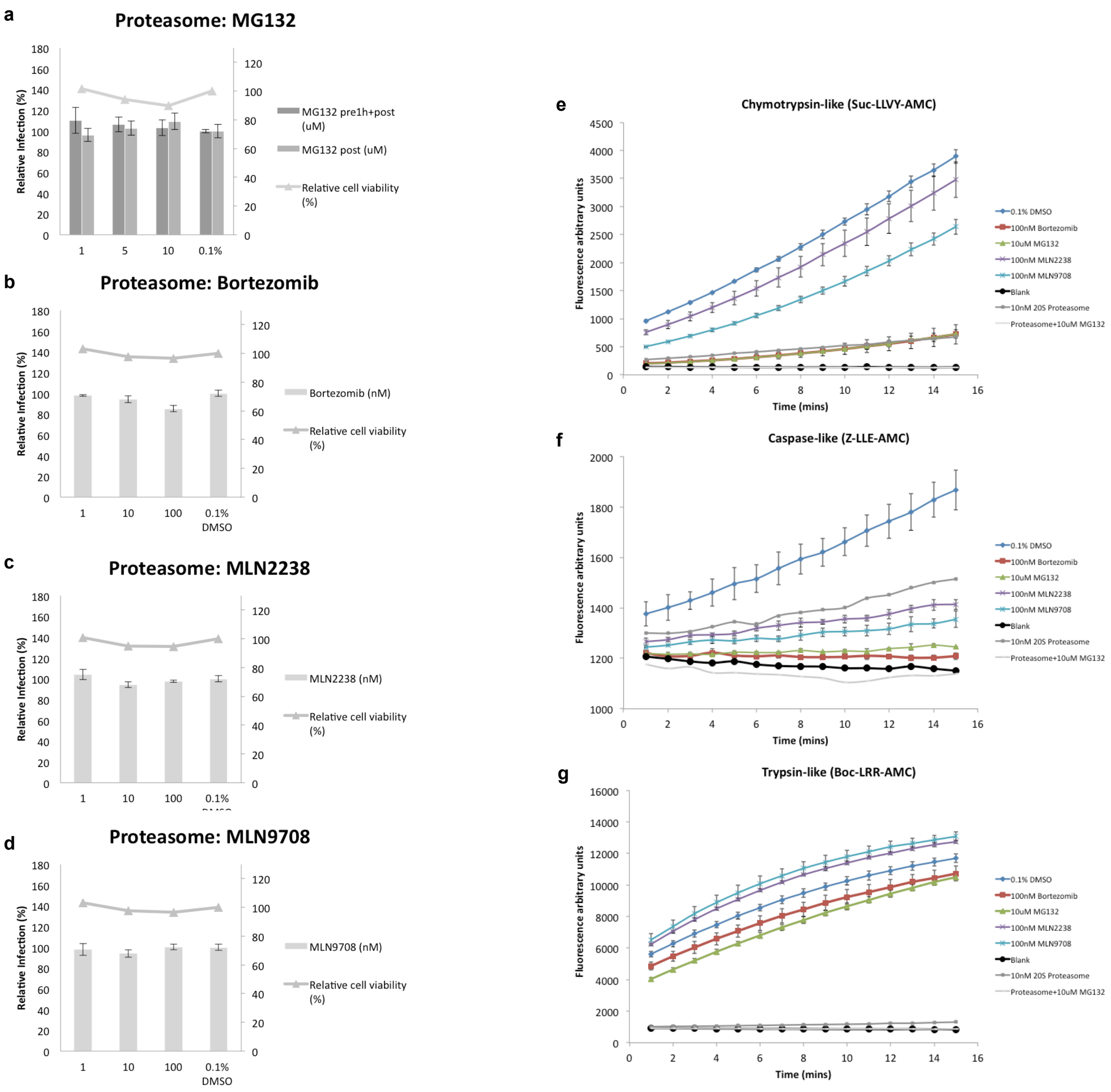

Supplementary Figure 6. Proteasome inhibitors have no impact on EV71 replication in RD cells. Mean relative infection rates of EV71-infected cells treated with **(a)** MG132, **(b)** Bortezomib, **(c)** MLN238 or **(d)** MLN9708 post-infection. Error bars show standard deviations. Data is obtained from two independent experiments and no significant difference was found when compared against vehicle control in two tailed, Student's T-Test. Proteasome activities upon inhibitor treatments were verified using a fluorometric proteasome activity assay kit (UBPbio). Inhibitor-treated RD cells were lysed and tested for (**e**) chymotrypsin-like, (**f**) caspase-like and (**g**) trypsin-like activities using the substrates Suc-LLVY-AMC, Z-LLE-AMC and Boc-LRR-AMC respectively. Assay controls include kit-

supplied 20S proteasome and MG132. Results shown are means of three independent experiments with error bars representing standard deviation.

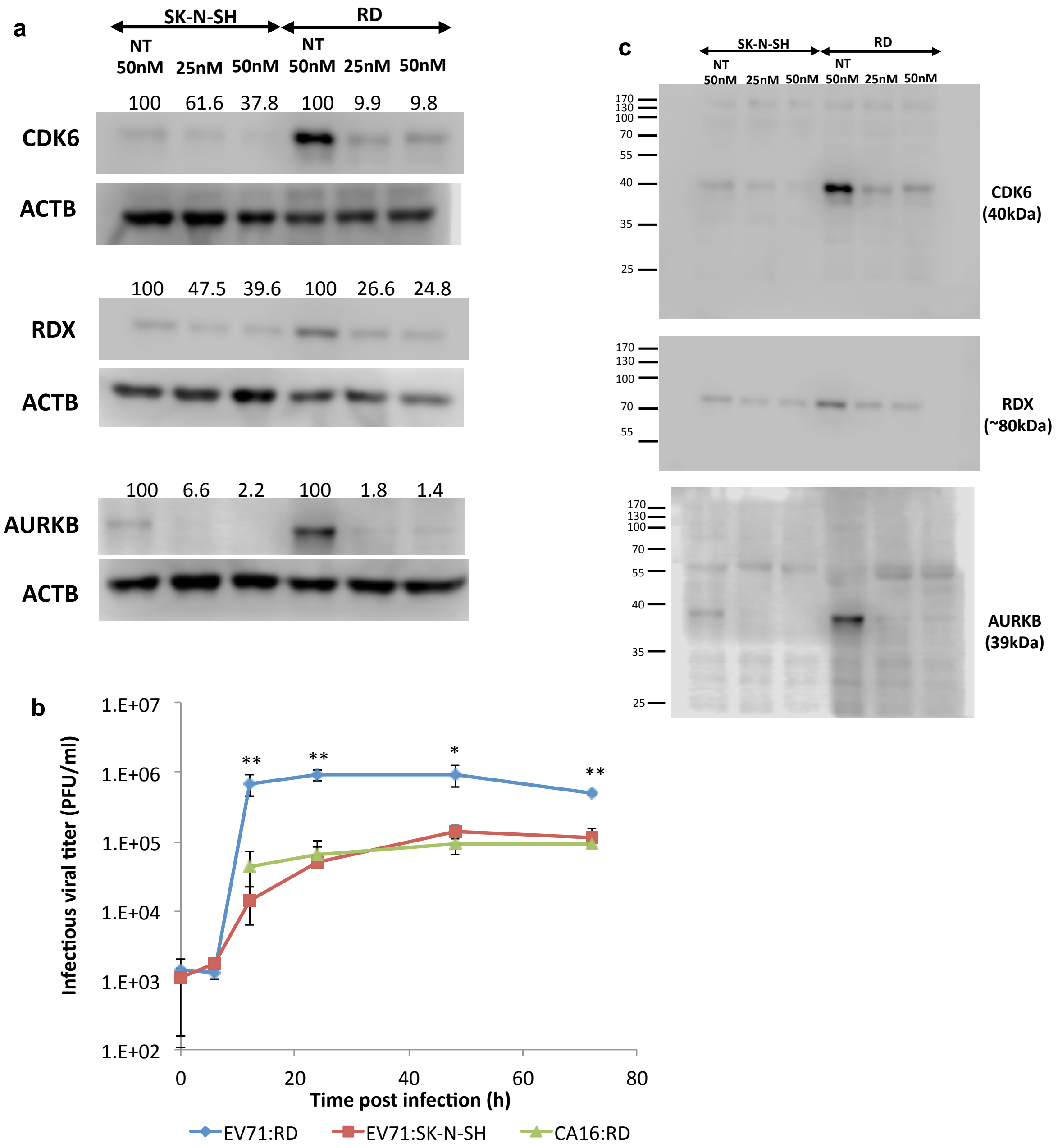

Supplementary Figure 7. siRNA knockdown efficiency and enteroviral replication efficiency in RD versus SK-N-SH cells. (**a**) SK-N-SH or RD cells were transfected with siRNAs against NT, CDK6, RDX or AURKB and knockdown was carried out for 3 days post transfection before lysis and analysis by SDS PAGE followed by Western blot. Densitometric analysis was carried out using Image Studio (Licor) and numbers shown are actin-normalized values for each band of interest, which are then presented as a percentage relative to each cell line's NT sample. (**b**) Growth kinetics of EV71 in RD cells and SK-N-

SH cells and CA16 in RD cells. Datapoints shown are mean infectious viral titer (PFU/ml) from three independent experiments with error bars showing standard deviation. Two-tailed Student's T-test was performed for EV71 titer at each timepoint in RD cells vs SK-N-SH cells with *$p<0.05$ and **$p<0.001$. (c) Uncropped scans of Western blots for the proteins of interests, CDK6, RDX and AURKB, with the markers representing indicated molecular weights (kDa). The RDX blot was excised from 55kDa onwards for simultaneous detection of beta actin (loading control).
